# Effectiveness of Ultra-High Irradiance Blue-Light-Emitting Diodes to Control *Salmonella* Contamination Adhered to Dry Stainless Steel Surfaces

**DOI:** 10.3390/microorganisms12010103

**Published:** 2024-01-04

**Authors:** Martha Minor, Luis Sabillón

**Affiliations:** 1 Department of Family & Consumer Sciences, New Mexico State University, Las Cruces, NM 88003, USA; 2 Center of Excellence in Sustainable Food and Agricultural Systems, New Mexico State University, Las Cruces, NM 88003, USA

**Keywords:** low-moisture foods, dry sanitation, food contact surfaces, enteric pathogens, blue light

## Abstract

Controlling *Salmonella* contamination in dry food processing environments represents a significant challenge due to their tolerance to desiccation stress and enhanced thermal resistance. Blue light is emerging as a safer alternative to UV irradiation for surface decontamination. In the present study, the antimicrobial efficacy of ultra-high irradiance (UHI) blue light, generated by light-emitting diodes (LEDs) at wavelengths of 405 nm (841.6 mW/cm^2^) and 460 nm (614.9 mW/cm^2^), was evaluated against a five-serovar cocktail of *Salmonella enterica* dry cells on clean and soiled stainless steel (SS) surfaces. Inoculated coupons were subjected to blue light irradiation treatments at equivalent energy doses ranging from 221 to 1106 J/cm^2^. Wheat flour was used as a model food soil system. To determine the bactericidal mechanisms of blue light, the intracellular concentration of reactive oxygen species (ROS) in *Salmonella* cells and the temperature changes on SS surfaces were also measured. The treatment energy dose had a significant effect on *Salmonella* inactivation levels. On clean SS surfaces, the reduction in *Salmonella* counts ranged from 0.8 to 7.4 log CFU/cm^2^, while, on soiled coupons, the inactivation levels varied from 1.2 to 4.2 log CFU/cm^2^. Blue LED treatments triggered a significant generation of ROS within *Salmonella* cells, as well as a substantial temperature increase in SS surfaces. However, in the presence of organic matter, the oxidative stress in *Salmonella* cells declined significantly, and treatments with higher energy doses (>700 J/cm^2^) were required to uphold the antimicrobial effectiveness observed on clean SS. The mechanism of the bactericidal effect of UHI blue LED treatments is likely to be a combination of photothermal and photochemical effects. These results indicate that LEDs emitting UHI blue light could represent a novel cost- and time-effective alternative for controlling microbial contamination in dry food processing environments.

## 1. Introduction

*Salmonella* is a leading cause of foodborne diseases worldwide [1]. In the United States alone, *Salmonella*-contaminated foods are responsible for more than 1 million illness cases each year [2]. *Salmonella* contamination is primarily associated with poultry, cattle, and their feeds; however, low-moisture foods (LMFs) have become an important transmission vehicle in recent years [3]. For instance, from 2007 to 2018, LMFs accounted for 21% of the *Salmonella* outbreaks investigated and reported in the U.S. by the Centers for Disease Control and Prevention (CDC) [4].

Several studies have shown that *Salmonella* has the ability to tolerate desiccation stress, allowing it to survive in LMFs and their processing environments for lengthy periods of time [5,6,7]. Furthermore, *Salmonella* cells subjected to dry conditions become more heat-tolerant [7]. This particular characteristic has clear implications for the safety of LMFs, as heat is a commonly used surface sanitation method in dry operations, making *Salmonella* an important emerging problem for this food sector. In general, *Salmonella* contamination events in LMFs have been traced to factors such as biofilm formation, inadequate sanitation, cross-contamination from equipment surfaces, and poor hygienic equipment design [8,9,10]. Moisture control is critically important in preventing the establishment and long-term persistence of *Salmonella* and other bacterial pathogens within LMFs manufacturing facilities [11]. Therefore, the use of wet cleaning and sanitation procedures is restricted in these operations due to the risk of introducing moisture into hard-to-reach, difficult-to-clean areas that can become thriving microbiological niches [12].

*Salmonella* outbreaks caused by LMFs not only underscore the difficulty of eradicating this pathogenic bacterium from dry processing environments, but also highlight the need to evaluate and incorporate novel dry surface sanitation technologies. Light-emitting diode (LED) technology is an emerging alternative to conventional mercury lamps for surface disinfection. LEDs are robust solid-state devices that emit monochromatic light by electroluminescence [13]. Light wavelengths in the ultraviolet region (200–400 nm) are widely used to inactivate foodborne pathogens, with UV-C light (200–280 nm) showing the strongest germicidal activity due to its ability to destroy DNA bonds and interrupt RNA transcription. However, UV-C LED irradiation has several critical limitations, including poor penetration depth, low emission intensity, the need to follow regulations established by major global regulatory authorities (e.g., U.S. Food and Drug Administration, Health Canada, and the European Union), and detrimental effects on human skin and eyes [14].

In recent years, LEDs emitting blue-colored wavelengths of the visible spectrum have been introduced and investigated as a safer alternative to UV irradiation. Several studies have shown that visible blue light (400 to 470 nm wavelength) exhibits broad-spectrum antimicrobial activity [15]. The main hypothesized mechanism of action involves the presence of naturally occurring photosensitizing molecules in microbial cells, such as flavins and porphyrins [15,16,17,18]. These endogenous photosensitizers absorb blue light, which induces excitation to the triple state and singlet oxygen generation [19]. Singlet oxygen molecules will react and produce other reactive oxygen spices (ROS), such as super-oxides, hydrogen peroxide, and hydroxyl radicals. The intracellular presence of ROS will result in irreversible oxidative damage to intracellular components such as DNA, proteins, and lipids [20,21,22,23]. Furthermore, a photothermal effect may also occur simultaneously, resulting in lethal temperature increases in microbial cells and the surrounding environment due to radiation heat transfer during treatment [24,25].

Studies on the surface decontamination potential of blue LEDs have largely been performed under conditions that simulate wet processing environments and with the use of exogenous photosensitizing solutions (e.g., curcumin and chlorophyllin) [26,27,28,29]. In contrast, few studies have tried to investigate the suitability of this light-based technology to control pathogen contamination in dry settings, including abiotic and biotic surfaces [13,24,30]. Moreover, the bactericidal efficacy of blue LEDs has generally been evaluated using low irradiance levels (<100 mW/cm^2^), requiring long treatment times (several hours) to achieve significant microbial reductions [31].

LED technology is rapidly becoming more efficient, and new prototypes are capable of emitting monochromatic blue light at an ultra-high-power density. Therefore, it was hypothesized that these enhanced blue LED devices would be more effective at reducing enteric pathogen contamination by combining photochemical and photothermal effects on microbial cells without exogenous photosensitizers. To prove this hypothesis, the following objectives were identified for this study: (i) to evaluate the efficacy of ultra-high irradiance (UHI) blue light treatments emitted by novel 405 and 460 nm LEDs to inactivate *Salmonella enterica* on dry stainless steel surfaces, (ii) to elucidate the photochemical and photothermal effects of UHI blue LEDs on *Salmonella* cells, and (iii) to examine the impact of a soil layer, using wheat flour as a model food soil system, on the antimicrobial efficacy of these treatments.

## 2. Materials and Methods

### 2.1. Bacterial Strains and Inoculum Preparation

Five *Salmonella enterica* serovars [Braenderup (ATCC 700136), Senftenberg (ATCC 8400), Typhimurium (ATCC 19585), Enteritidis (ATCC 13076), Montevideo (ATCC BAA-710)] were used in this study. These *Salmonella* serovars were chosen for this experiment based on their role in foodborne outbreaks associated with low-moisture products. All bacterial strains were obtained from the American Type Culture Collection (ATCC; Manassas, VA, USA) and propagated according to ATCC’s recommendation. After propagation, all cultures were stored individually at −80 °C in cryogenic vials containing 20% (*v*/*v*) sterile glycerol (Fisher Scientific; Fisher BioReagents™; Waltham, MA, USA) for long-term preservation. All cultures were re-activated individually by scraping the frozen broth with a sterile loop (10 μL) and transferring it into 9 mL Tryptic Soy Broth (TSB; BD Difco™ Bacto™, Sparks, MD, USA), followed by incubation at 37 °C for 24 ± 2 h. This overnight culture was used to prepare the working stock plates for upcoming experiments. To obtain isolated colonies, a loopful (1 μL) of the overnight bacterial suspension was streaked onto Tryptic Soy Agar plates supplemented with 0.6% (*w*/*w*) Yeast Extract (TSA-YE; CRITERION™, Hardy Diagnostics, Santa Maria, CA, USA) and incubated at 37 °C for 24 ± 2 h. Working stock plates were then wrapped with Parafilm™ M (PM-996; Bemis™; Bemis Manufacturing Company; Sheboygan Falls, WI, USA) and stored in a refrigerator at 4 °C. These plates were used within 30 days to prepare the inoculum.

One isolated colony from the working stock plate of each strain was transferred, individually, to 9 mL TSB tubes and incubated at 37 °C for 24 ± 2 h. A bacterial lawn was created for each strain, by spreading 0.1 mL of the overnight broth culture onto TSA-YE agar plates, and incubated for 24 ± 2 h at 37 °C. The lawns were then harvested by adding 6 mL of 0.1% buffered peptone water (BPW; Difco™; Sparks, MD, USA) to each agar plate and scraping the bacterial cells using an L-shaped spreader. Using serological pipets, equal amounts of cell suspensions were transferred to a 50 mL sterile conical tube to create a five-serovar cocktail of *S. enterica*. Bacterial cells were then harvested by centrifugation (4000× *g*/4 °C for 8 min) and the resulting pellet washed twice with 10 mL 0.1% BPW. Cleaned pellets were finally resuspended in 4 mL of sterile distilled water.

### 2.2. Preparation and Inoculation of Stainless Steel Surfaces

Disc coupons (diameter: 1.27 cm) made of stainless steel (type 304) were used in this study (BioSurface Technologies Corporation; Bozeman, MT, USA). Coupons were cleaned, sterilized, and inoculated, as described by Minor and Sabillón [24]. Briefly, sterile coupons were spot inoculated with 70 µL of the *Salmonella* cocktail or with 70 µL of a *Salmonella*-contaminated flour slurry to achieve an approximate concentration of 9.0 and 8.0 log CFU/cm^2^, respectively. All-purpose wheat flour was used as a model food soil system, at a concentration of 0.25 g/mL (0.025%), to evaluate the impact of organic matter on the effectiveness of blue light treatments. Inoculated coupons were allowed to air dry inside the biosafety cabinet for 3 h, with the fan running, to allow for moisture evaporation and the transition of cells from the planktonic to the sessile state.

### 2.3. Treatment Approach

#### 2.3.1. The Light-Emitting Diode (LED) System

**Figure 1:**
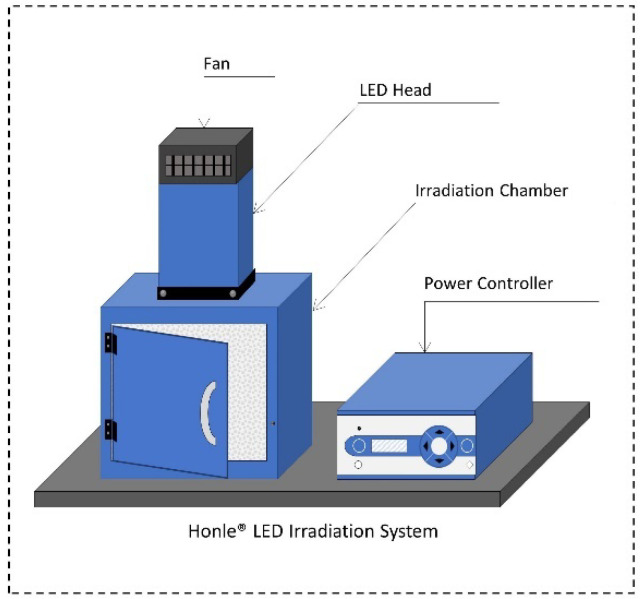
Schematic view of the Honle^®^ LED Irradiation System.

The LED irradiation system (Honle^®^, LED Cube 100 IC) was acquired from Panacol-USA, Inc. (Torrington, CT, USA). The LED system consisted of (i) an electronic power controller (Part #: 81621), (ii) an irradiation chamber with a reflective interior wall structure (Dimensions: 18 cm × 18 cm × 18 cm [H × W × D]; Part #: 81630), and (iii) Honle^®^ LED heads emitting light at 405 nm (Part #: 85689) and 460 nm (Part #: 85684). LED lamps were mounted on top of the irradiation chamber and connected to the power controller with LED power set to 100%, as shown in Figure 1. Both the chamber and light heads were equipped with fans to avoid overheating during treatments. The radiant power per unit of surface area (i.e., irradiance [mW/cm^2^]) for each LED head was determined using a hand-held Honle^®^ UV-meter (Part #: 86001) coupled with a LED spectrum surface sensor (FS LED D2, Part #: 80434) positioned at 5 cm from the LED head.

#### 2.3.2. Ultra-High Irradiance (UHI) Monochromatic Blue Light Treatments

Following the drying period (3 h), an aluminum pan containing four inoculated coupons was placed inside the irradiation chamber, leaving a 5 cm gap between the light emitter head and coupon surfaces. Subsequently, *Salmonella* sessile communities on metal coupons were irradiated with 405 and 460 nm LEDs under equivalent energy doses, achieved by varying the treatment times at 100% LED power (Table 1). In a series of preliminary experiments, inoculated coupons were subjected to blue light irradiation with a 460 nm LED (100% power) for various exposure times to determine the best treatment regimen that would achieve a minimum of a 5-log reduction in *Salmonella* counts, thereby meeting and/or exceeding the performance standards established by the US-FDA for pathogen control interventions. Based on preliminary data, it was established that irradiating unsoiled surfaces with a 460 nm LED for 18 min (664 J/cm^2^) would exceed those FDA performance standards. The minimum exposure time (i.e., 18 min) was then divided into 6 min increments (0, 6, 12, and 18 min) to show incremental gains in *Salmonella* reduction. Subsequently, the exposure times for the 405 nm LED treatments were calculated to provide equivalent energy doses, facilitating a fair comparison between the two light wavelengths. The same approach was applied to flour-coated surfaces; however, longer exposure times were necessary to achieve a substantial *Salmonella* reduction.

The total radiant energy emitted by the 405 and 460 nm LEDs at 100% power was 842 and 615 mW/cm^2^, respectively. The energy dose (i.e., fluence [J/cm^2^]) received by coupon surfaces per unit area was calculated by multiplying the radiant energy (mW/cm^2^) and exposure time (seconds). The surface temperature of metal coupons was captured before and immediately after UHI light treatments with a thermographic camera (FLIR ONE Pro; Teledyne FLIR, Wilsonville, OR, USA). Coupons without light treatment were used as controls. Two technical replicates (i.e., coupons) were used in each treatment, and triple independent experiments were conducted for each treatment combination.

### 2.4. Microbiological Analysis

#### 2.4.1. Determination of Viable *Salmonella* Cells

To enumerate the surviving *Salmonella* population after UHI light treatments, treated coupons were aseptically transferred into 50 mL sterile conical tubes containing 10 mL of full-strength BPW and 2 g of glass beads (2 mm diameter; BioSpec Products, Inc., Bartlesville, OK, USA). Tubes were then vortexed at high speed (3000 rpm) for 2 min to assure complete detachment of bacterial cells. This method had been previously validated in our laboratory to maximize cell recovery from coupon surfaces. The detached bacterial suspension was 10-fold serially diluted with 0.1% BPW and appropriate dilutions spread-plated in duplicate on modified TSA plates containing an H_2_S indicator system. TSA was supplemented with 0.6% (*w*/*v*) yeast extract, 0.03% (*w*/*v*) sodium thiosulfate (Sigma-Aldrich, Inc.; St. Louis, MO, USA), and 0.05% (*w*/*v*) ammonium ferric citrate (Sigma-Aldrich, Inc.) to allow not only the recovery of stressed/sub-lethally injured *Salmonella* cells, but also the differentiation of these cells from potential microbial contaminants. Plates were then incubated at 37 °C for 24 ± 2 h prior to enumeration, and only those colonies that had a dark center were counted as *Salmonella* cells. The final cell counts were expressed in Log CFU/cm^2^.

In the event that a coupon yields plate counts below the limit of detection (<1 log CFU/cm^2^), a molecular PCR-based pathogen detection system (GENE-UP^®^; bioMérieux, Inc., Salt Lake City, UT, USA) was ready to be used to corroborate the absence of *Salmonella* spp. For this purpose, after serial dilution, the conical tubes containing the remaining detached bacterial suspensions in full-strength BPW were placed in an incubator at 41 °C for 24 ± 2. Tubes with turbid broth were pulled from the incubator and the GENE-UP^®^ *Salmonella* 2 assays were performed according to the manufacturer’s directions. In addition, turbid broths were sub-cultured on Xylose-Lysine-Deoxycholate (XLD Agar, Remel™, Thermo Fisher Scientific, Inc., Waltham, MA, USA) agar plates for confirmation of *Salmonella* presence based on typical colony morphology, after incubation at 37 °C for 24 ± 2 h.

#### 2.4.2. Determination of Oxidative Stress in *Salmonella* Cells

The quantification of intracellular reactive oxygen species (ROS) in *Salmonella* cells was performed using a general oxidative stress indicator (5(6)-carboxy-2′,7′-dichlorodihydroflourescein diacetate (Carboxy-H2DCFDA) (Invitrogen, Eugene, OR, USA), according to a procedure described by Minor and Sabillón [24]. Fluorescence values were measured using a fluorometer (Quantus; Promega Corporation, Madison, WI, USA) with excitation and emission wavelengths of 495 nm and 525 nm, respectively. The intracellular concentration of ROS was expressed as relative fluorescence intensity in arbitrary units (AU).

### 2.5. Data Analysis

Triplicate data were analyzed with statistical package software SAS version 9.4 (SAS Institute, Cary, NC, USA), using a two-way analysis of variance (ANOVA) to compare the changes in *Salmonella* load on coupon surfaces in response to light wavelength and energy dose. ANOVAs were performed by using the GLIMMIX procedure of SAS. The LSMEANS procedure was used to calculate the mean and standard error of the various treatment replicates. Tukey’s multiple comparison test was used to determine significant differences in microbial reductions among treatments. All statistical analysis was performed with a significance level of *p* ≤ 0.05.

## 3. Results

### 3.1. Efficacy of UHI Blue Light Treatments against Salmonella on Clean Stainless Steel Surfaces

**Figure 2:**
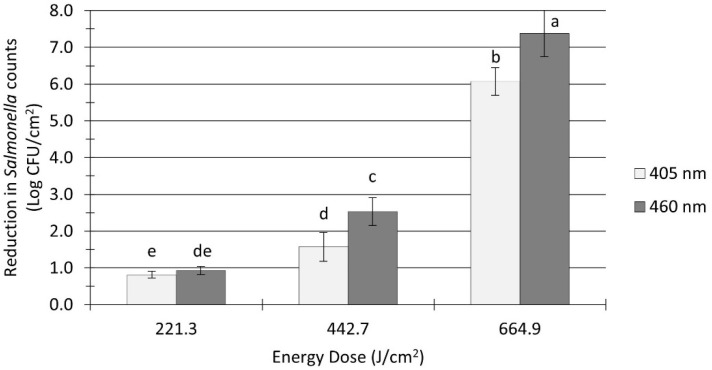
Reduction in *Salmonella* spp. counts in unsoiled stainless steel coupons after ultra-high irradiance light treatments using 405 and 460 nm LEDs. Coupons were treated at 5 cm distance from the LED head. Log reduction values (Log CFU/cm^2^) are expressed as mean ± standard deviation (*n* = 6). Values with different letters are significantly different from one another (*p* ≤ 0.05).

The initial *Salmonella* sessile population on clean (no added soil) stainless steel coupons was 9.5 ± 0.1 log CFU/cm^2^ before the application of treatments. As illustrated in Figure 2, the *Salmonella* population was significantly reduced by all UHI blue light treatments, with reduction levels ranging from 0.8 to 7.4 log CFU/cm^2^. The treatment energy dose had a significant effect on the reduction in *Salmonella* counts (*p* < 0.0001). For instance, increasing the dose of 405 nm light treatments from 221 to 665 J/cm^2^ reduced *Salmonella* counts from 0.8 to 6.1 log CFU/cm^2^. The type of wavelength also had a significant effect on *Salmonella* inactivation (*p* < 0.0001). Overall, 460 nm light treatments were more effective at reducing *Salmonella* counts (average reduction: 3.6 log CFU/cm^2^) on stainless steel surfaces when compared to 405 nm light treatments under equivalent energy dose (average reduction: 2.8 log CFU/cm^2^).

### 3.2. Effect of a Soil Layer on Salmonella Inactivation by UHI Blue Light Treatments

**Figure 3:**
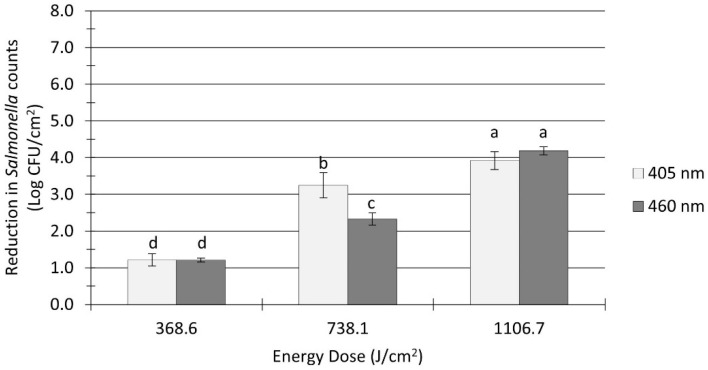
Reduction in *Salmonella* spp. counts in soiled (flour-coated) stainless steel coupons after ultra-high irradiance light treatments using 405 and 460 nm LEDs. Coupons were treated at 5 cm distance from the LED head. Log reduction values (Log CFU/cm^2^) are expressed as mean ± standard deviation (*n* = 6). Values with different letters are significantly different from one another (*p* ≤ 0.05).

The pretreatment *Salmonella* population in flour-coated stainless steel coupons was 8.1 ± 0.1 log CFU/cm^2^. The *Salmonella* inactivation levels on soiled surfaces ranged from 1.2 to 4.2 log CFU/cm^2^, as shown in Figure 3. A significant effect (*p* < 0.0001) of organic soil on the inactivation efficacy of blue LEDs was observed, as higher light energy doses were required to achieve notable microbial reductions. For example, treatments with 405 nm at an energy dose of 738 J/cm^2^ reduced *Salmonella* counts by 3.3 log CFU/cm^2^ on soiled surfaces (Figure 3), while a much lower dose of 665 J/cm^2^ applied to clean surfaces resulted in twice as much reduction (6.1 log CFU/cm^2^; Figure 2). In general, the reduction levels of *Salmonella* count on soiled surfaces were conditioned by the treatment energy dose and the light wavelength. Increasing the energy dose from 369 to 738 J/cm^2^ and above produced significant (*p* < 0.0001) further reductions in counts (Figure 3). Overall, 405 nm light treatments (average reduction: 2.8 log CFU/cm^2^) were significantly (*p* = 0.027) more effective than 460 nm treatments (average reduction: 2.6 log CFU/cm^2^) at reducing the number of *Salmonella* cells on soiled surfaces; however, the observed differences in microbial reductions were so small that they may not be practically relevant from a microbiological standpoint.

### 3.3. Quantification of Oxidative Stress in Salmonella Cells

**Figure 4:**
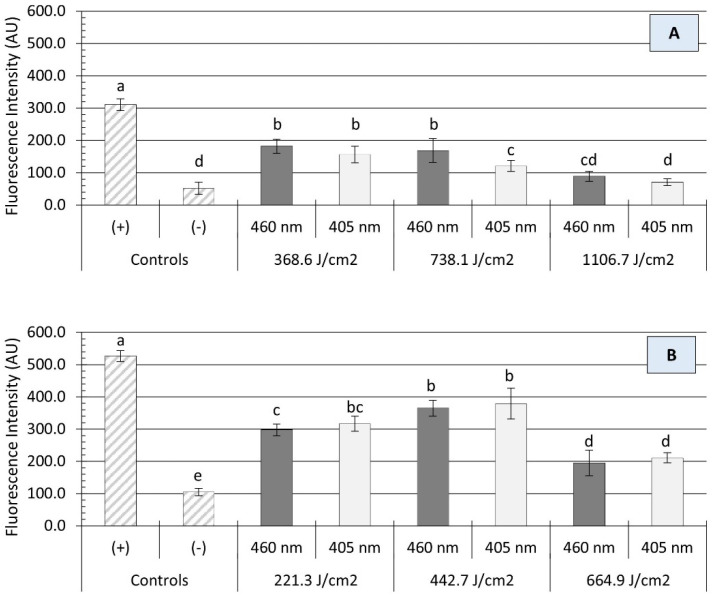
Production of intracellular reactive oxygen species (ROS) in *Salmonella* cells after the application of ultra-high irradiance blue light (405 and 460 nm LEDs) treatments on dry stainless steel surfaces with (**A**) and without (**B**) the presence of organic matter (wheat flour). The data are presented as mean ± standard deviation (*n* = 6). Fluorescence intensity values, within the same figure panel, with different letters are significantly different from one another (*p* ≤ 0.05). Negative control (−): untreated *Salmonella* cells; positive control (+): *Salmonella* cells treated with 200 mM H_2_O_2_.

The bactericidal activity of blue light is primarily associated with the excitation of intracellular photosensitizing molecules, resulting in the production of reactive oxygen spices (ROS), such as singlet oxygen (O_2_), hydrogen peroxide (H_2_O_2_), and hydroxyl radicals (HO^−^). High concentrations of ROS may damage microbial cells by oxidizing numerous molecular targets, including proteins, lipids, and nucleic acids [32]. In the present study, irradiation with either a 405 or 460 nm light source caused a significant production of ROS within *Salmonella* cells, as measured by Carboxy-H2DCFDA staining (Figure 4). In general, the intracellular generation of ROS was not significantly influenced by the light wavelength (*p* > 0.05) but rather by the treatment energy dose (*p* < 0.05) and the presence of organic matter (*p* < 0.0001). On clean surfaces, illumination with each wavelength produced an energy-dose-dependent effect on the amount of oxidative stress endured by *Salmonella* cells. For instance, a significant increase in ROS production was noted in *Salmonella* cells when the treatment energy level increased from 221 to 443 J/cm^2^, followed by a significant decline at a higher dose of 665 J/cm^2^ (Figure 4B). The presence of organic matter (i.e., wheat flour) had a protective effect on *Salmonella* cells against oxidative stress, as a significant reduction (*p* < 0.001) in ROS generation was observed (Figure 4A). Despite increases in energy doses, the average fluorescence intensity of *Salmonella* cells detached from soiled surfaces remained relatively unchanged. In fact, no significant differences in fluorescence intensity were observed between 1107 J/cm^2^ treatments and negative controls (*p* > 0.05).

### 3.4. Temperature Changes on Stainless Steel Surfaces during UHI Blue Light Treatments

**Figure 5:**
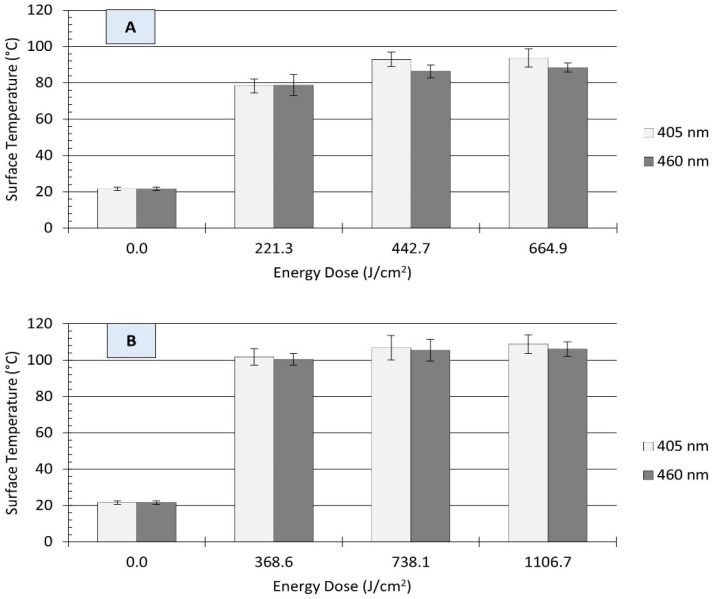
Surface temperature of unsoiled (**A**) and soiled/flour-coated (**B**) stainless steel (type 304) coupons as affected by ultra-high irradiance blue light treatments at 405 and 460 nm. Error bars denote ± standard deviation. Surface temperature was captured with a thermographic camera immediately after treatment.

Light-based antimicrobial interventions may also induce thermal damage to microbial cells when light energy is converted into heat energy by irradiated surface materials. This heat energy can act synergistically with the generated intracellular ROS, thus improving the antimicrobial efficacy of blue light treatment. In the present study, UHI light treatment resulted in substantial increases in the surface temperature of stainless-steel coupons, as depicted in Figure 5. On clean surfaces, illumination with each wavelength produced an energy-dose-dependent effect on the amount of temperature increase. For example, irradiation with 405 nm light at 221 J/cm^2^ increased the surface temperature of coupons from 22 ± 1 to 78 ± 4 °C, while treatments with a higher energy at 665 J/cm^2^ resulted in a much higher temperature of 94 ± 5 °C (Figure 5A). A similar trend was observed for those treatments with 460 nm LEDs. In general, a direct relationship was observed between treatment energy dose and the surface temperature of clean stainless steel coupons.

The presence of organic matter (i.e., wheat flour) reduced the energy-dose-dependent effect on surface temperature. For instance, treatments with 369 J/cm^2^ increased the surface temperature of soiled stainless steel surfaces from 22 ± 1 to 101 ± 1 °C (ΔT: 79 ± 1 °C), regardless of the light wavelength. Raising the treatment energy dose to 738 or 1107 J/cm^2^ resulted in only small temperature increments, with maximum surface temperatures ranging from 105 to 109 °C (Figure 5B). Previous studies have suggested that temperature increase due to LED treatments is wavelength-dependent [25]. In the present study, differences in the surface temperature of stainless steel coupons were observed between 405 and 460 nm treatments under equivalent energy dose; however, these differences were small and may not be relevant for microbial inactivation.

## 4. Discussion

Despite advances in food safety measures, foodborne illness outbreaks and recalls linked to low-moisture products continue to occur. Therefore, the development and implementation of new antimicrobial interventions are essential to improve the microbiological safety of dry food processing environments. In recent years, the blue-light-induced inactivation of microorganisms has drawn attention not only as a safer alternative to UV-C irradiation, but also as a promising intervention to combat antimicrobial resistance in the farm-to-table continuum.

Previous studies have shown that blue-colored wavelengths in the visible light spectrum have tremendous potential for surface disinfection. For instance, shorter wavelengths of blue light such as 405–425 nm have been shown to reduce *Cronobacter sakazakii*, *E. coli* O157:H7 and *Salmonella* spp. counts by 1 to 2 logs in liquids and dry foods [33,34], while longer blue light wavelengths (453–470 nm) have achieved 0.9 to 3.2 log reductions in *Salmonella* populations inoculated in dry pet food and wheat flour [13,25,35]. However, these research studies have been performed with low-irradiance LEDs (<100 mW/cm^2^), which require longer exposure times (several hours) to accomplish targeted microbial reductions [36]. In comparison to LEDs emitting low-irradiance blue light, UHI blue LEDs can be used to deliver treatments that can achieve a combination of photothermal and photochemical effects on bacterial cells, thereby improving inactivation rates [24]. Therefore, the present study focused on understanding the inactivation efficacy of new LED technology, using UHI short (405 nm; 842 mW/cm^2^) and long (460 nm; 615 mW/cm^2^) blue light wavelengths against a five-serovar cocktail of *Salmonella enterica* on dry stainless steel surfaces.

As a performance standard, the U.S. Food and Drug Administration requires that the control processes, such as heat or UV light, used to reduce pathogen burden achieve a minimum of 5-log reduction in the targeted microorganism [37]. In this study, the treatment of unsoiled stainless steel surfaces with UHI 405 or 460 nm LEDs at 665 J/cm^2^ resulted in reductions of >6.0 log CFU/cm^2^ in *Salmonella* counts (Figure 2), surpassing FDA-recommended reduction levels. Similar research studies have also demonstrated the effectiveness of UHI blue LEDs to inactivate vegetative forms and spores of several pathogens. For instance, the efficacy of these UHI blue LEDs against *E. coli* O157:H7 was recently documented by Minor and Sabillón [24], who reported inactivation levels ranging from 2.0 to >8.0 log CFU/cm^2^ on dry metal surfaces. Likewise, Lang, et al. [36] found that short-time treatments (below 10 min) with a UHI 405 nm LED induced >4.5 log reduction in *S. cerevisiae* counts inoculated in several food contact surface materials. A study by Thery et al. [38] has shown the sporicidal efficiency of UHI blue LED treatments against spores of *B. cereus*, *S. cerevisiae*, and *Penicillium* spp., with reduction levels ranging from 0.8 to 4.2 log CFU/mL. In comparison, the antimicrobial effectiveness of UV-C light under dry conditions appears to be limited. For example, LED treatments with UV-C light at 275 nm for 60 min (60.1 J/cm^2^) resulted in 1.1 log reduction in *Salmonella* counts in wheat flour [13]. Similarly, Cheon et al. [39] reported reduction levels of 0.2 and 0.3 log CFU/g in *E. coli* O157:H7 and *S.* Typhimurium counts, respectively, on powdered red pepper after treatment with UV-C irradiation at 20.4 kJ/m^2^ for 10 min. On dry stainless steel surfaces, Bae and Lee [40] showed that UV-C (253 nm) treatment at 0.24 mW/cm^2^ for 3 h could reduce *S.* Typhimurium, *L. monocytogenes*, and *S. aureus* by as much as 3.1, 2.2, and 2.7 log CFU/coupon, respectively.

The mechanism of microbial inactivation by visible blue light is primarily attributed to the irreversible damage to various molecular constituents of cells caused by the formation of highly reactive oxygen ions (ROS) during electron/energy transfer reactions from light to photosensitizing molecules (e.g., flavins and porphyrins) [32,41]. Several studies have demonstrated that phospholipids and proteins in cellular envelopes are major targets of ROS during blue light irradiation, significantly impairing membrane structure and function [41,42]. Furthermore, although DNA does not appear to be the primary target, previous studies by Grinholc et al. [43] and Kim, Bang, and Yuk [44] confirmed that bacterial genomic DNA can be oxidized by ROS, thereby enhancing the antimicrobial properties of blue light. In fact, guanine bases of DNA are particularly susceptible to oxidation by singlet oxygen [45]. Also, it is known that charged porphyrin molecules can bind to and interact with cellular DNA in different ways, thereby interfering with DNA replication, recombination, and repair [41]. In addition to the inactivation induced by photochemical effects, when delivered at high energy densities, blue LEDs may also induce photothermal damage to microbial cells [36,46], thereby contributing to pathogen inactivation. Due to the damage to multiple cellular components, it may be difficult for bacteria to develop resistance to blue-light-based treatments [47], which makes it a promising intervention to improve food safety.

In this study, UHI blue light treatments were found to increase both ROS levels in *Salmonella* cells and the temperature of irradiated surfaces in an energy-dose-dependent manner (Figure 4 and Figure 5). Since ROS and temperature changes were measured at the same time point after blue light exposure in this experiment, it is difficult to establish whether *Salmonella* inactivation was directly caused by a synergistic or additive interaction between these two photoinduced phenomena. Nonetheless, the decrease in ROS concentration observed in *Salmonella* cells after a continuous long-term exposure (>665 J/cm^2^) to UHI blue light may suggest the existence of a threshold, after which photothermal effects govern microbial inactivation mechanisms. This hypothesis needs to be verified by further experiments in the future. The findings reported in this study are in line with those of previous studies by Minor and Sabillón [24] and Prasad, Gänzle, and Roopesh [35], who found significant oxidative stress in dehydrated *E. coli* O157:H7 and *Salmonella* Typhimurium cells, respectively, after exposure to blue light LEDs.

Earlier studies have also documented the photothermal impact of violet/blue (395–470 nm) LED treatments on food dehydration and microbial reduction [13,25,30,48,49], with some authors highlighting the role of the thermal properties of foods and contact surface materials on microbial inactivation rates [24,36]. The temperature rise due to the application of UHI blue light observed in this study (Figure 5) is a dissipative phenomenon that cannot maintain itself unless a constant source of energy is supplied, and the resulting magnitude (temperature rise) depends on the radiant energy dose and thermophysical properties of the material, such as the specific heat capacity (cp), thermal conductivity, and thermal diffusivity. For instance, the cp of wheat flour (1.66 kJ/kg °C) is four times higher than that of stainless steel (0.461 kJ/kg °C) [24], thus requiring elevated energy doses to increase its temperature. Surface materials with high thermal conductivity (e.g., stainless steel vs HDPE) could effectively undergo fairly uniform bacterial inactivation due to efficient heat transmission.

The notable temperature increases observed on treated stainless steel surfaces during the UHI blue LED treatments are not superior to those achieved by other heat-based sanitation technologies commonly used in low-moisture food processing operations, such as dry heat and superheated steam (SHS). For instance, treatment temperatures of 135 °C represent the low spectrum of SHS applications, while temperatures >160 °C are often applied to soiled surfaces [50]. Treatments with dry heat are typically performed at temperatures ranging from 80 to 100 °C, requiring long exposure times to cause notable microbial inactivation. For example, dry heat (80 °C) caused a reduction of 4.0 log CFU/cm^2^ after 15 h on *Listeria innocua* sessile cells on aluminum [51]. Almatroudi et al. [52] evaluated the effect of dry heat on a *Staphylococcus aureus* biofilm formed on polycarbonate coupons. After dry heat treatments of 80 °C for 1 h and 100 °C for 10 min, the authors reported reductions of 0.1 and 0.9 log CFU/cm^2^, respectively. In comparison to these traditional heat treatments, the results obtained in this study suggest that UHI LED technology could be used as an effective tool to further address microbiological risks in dry food operations.

Compared to UV light, blue visible light offers a series of advantages, including lower energy and longer wavelengths, providing a deeper penetration capacity [17]. Nevertheless, this penetration advantage may be limited by the presence of organic matter on target surfaces. In this study, a reduction in the antimicrobial efficacy of UHI blue LED treatments was observed when applied to flour-coated stainless steel surfaces (Figure 3). In addition, a significant decline in ROS generation within *Salmonella* cells was also noticed in the presence of organic matter (Figure 4A). These findings confirm those of previous studies by Ziuzina et al. [53] and Minor and Sabillón [24], suggesting that flour particles may have provided a shadowing/shielding effect, thus protecting *Salmonella* cells against light-induced oxidation and heat stress. This protective effect became apparent during preliminary experimentation in this study, where it was noted that longer treatment times (i.e., higher energy doses) were required to achieve microbial inactivation levels comparable to those obtained on clean stainless steel surfaces. In general, our findings suggest that, in the presence of organic matter, the mechanism of microbial inactivation by UHI blue LEDs is more likely to be photothermal rather than photochemical. Therefore, cleaning/removing soil deposits from food contact surfaces before treatment is crucial to ensure the adequate exposure of bacterial cells to LED light, which will increase the success rate of light-based antimicrobial interventions. In addition, factors such as surface roughness and hydrophobicity may also impact the effectiveness of LED treatments. According to Kim and Kang [54], surface hydrophobicity had a significant effect on bacterial stacking arrangements, causing undesirable shading effects and uneven exposure to UV-C LED irradiation. Therefore, given the wide range of surface materials used in the food industry, LED treatments that combine photochemical and photothermal antimicrobial mechanisms will be more effective and have a better chance of gaining relevance in the food industry. Consequently, a validation study to assess the effectiveness of UHI blue LED treatments under real-world dry food processing settings is warranted to explore the practical applications and scalability of this technology.

LEDs emitting blue light (405 to 470 nm) have emerged as a safer alternative to conventional ultraviolet radiation for surface decontamination. Exposure to UV radiation (<315 nm [UV-B and UV-C]) has been associated with skin cancer and other cell mutations [55]. Unlike UV radiation, blue light is innocuous on the skin; however, prolonged exposure to blue light illumination may cause phototoxicity toward human corneal and conjunctival epithelial cells [56]. Therefore, the adoption of blue LEDs for surface sanitation in food processing environments must be accompanied by robust safety standards and the use of appropriate personal protective equipment, such as safety glasses. Establishing Best Management Practices and Standards will also help develop a framework for the food industry that would provide guidance in the design, installation, and adoption of LED technology [57].

## 5. Conclusions

This study provided insight into the potential application of LEDs emitting monochromatic blue light (405 or 460 nm) at ultra-high irradiance (UHI) levels for the control of microbial contamination in dry food processing environments. The *Salmonella* population on clean and soiled stainless steel (SS) surfaces was significantly reduced by all UHI blue light treatments evaluated in this study. However, the presence of organic matter reduced the efficacy of these treatments against *Salmonella* contamination, thus highlighting the importance of cleaning surfaces before the application of light-based antimicrobial interventions. The treatment energy dose had a significant effect on *Salmonella* inactivation levels. Blue LED treatments triggered a significant generation of ROS within *Salmonella* cells, as well as a substantial temperature increase in SS surfaces. The results obtained in this study suggest that the mechanism of microbial inactivation by UHI blue light is due to a combination of oxidative and heat stress. When applied at UHI levels, blue LEDs could overcome the need for prolonged treatment times and the use of exogenous photosensitizing solutions, which are two major obstacles for the adoption of this technology in dry food operations. Overall, LEDs emitting monochromatic short (405 nm) or long (460 nm) blue light wavelengths with ultra-high intensity showed promising results as a viable and time-effective dry sanitation method. Future studies are warranted to assess the efficacy of UHI blue light against bacterial endospores and dry-surface biofilms. The impact of surface characteristics, such as roughness and hydrophobicity, on treatment effectiveness also needs further investigation.

## Figures and Tables

**Table 1:** Ultra-high irradiance blue light treatments applied to unsoiled and flour-coated stainless steel surfaces using 405 and 460 nm LEDs.

Organic Soiling ^a^	Light Wavelength	Treatment Intensity		
		Irradiance (mW/cm^2^) ^b^	Treatment Time (min)	Energy Dose (J/cm^2^)
None	405 nm	842	4.4	221.3
			8.8	442.7
			13.2	664.9
	460 nm	615	6.0	221.4
			12.0	442.7
			18.0	664.1
All-Purpose Wheat Flour	405 nm	842	7.3	368.6
			14.6	738.1
			21.9	1106.7
	460 nm	615	10.0	368.9
			20.0	737.9
			30.0	1106.8

^a^ None: no soil added to stainless steel surfaces; all-purpose wheat flour: used to emulate the presence of organic soiling on the metal surface. ^b^ Irradiance level emitted by LEDs at 100% power as determined by a UV-meter/LED spectrum surface sensor at a distance of 5 cm from the light emitting head.

## Data Availability

The data that support the findings of this study are available from the corresponding author upon reasonable request.
